# Foreign Body in the Right Bronchus; Tracheotomy; Removal and Recovery

**Published:** 1860-11

**Authors:** John M. Adler

**Affiliations:** Davenport, Iowa


					﻿Art. II. — Cases, with Remarks, from the Surgical Clinic of the
Jefferson Medical College.—Service of Professor Gross.
Reported by Emil Fischer, M.D., of Philadelphia.
I.— Two Cases of Spina Bifida; Treatment by Injections of Iodine;
Death.
Case 1.—James Grove, a healthy infant, aged eleven weeks, was ad-
mitted into the Clinic of the Jefferson Medical College, in October, 1859,
on account of a congenital tumor over the lumbar region. The swelling,
which at the time of birth had been as large as a walnut, had gradually
increased to its present size, which was that of a large orange. It was of
globular form, narrow at its base, and was situated in the median line,
over the spinous processes of the last lumbar vertebrae The integuments
covering it were much attenuated, particularly at the top of the tumor,
where they seemed ready to burst; they were reddened by capillary in-
jection, the discoloration being most intense at the lower part of the
tumor and around its margin. The tumor was translucent, and distinctly
fluctuating; compression did not decrease its bulk; no impulse was im-
parted to the finger, placed on the fontanel, when the tumor was pressed,
nor did the pressure produce any pain or convulsions.
The mother stated that the child had been born at full term, and had
always enjoyed good health; it had perfect use of both lower extremities,
and its urine and feces were under the power of the will. The head was
of natural size, and there was no club-foot, or other deformity. The skin
over the tumor had been, at some previous time, the seat of superficial
ulceration. Professor Gross pronounced the case to be one of spina
bifida, and taking into consideration that pressure upon the tumor did
not produce any effect upon the nervous centres, and that the functions
of the body were not disturbed to any appreciable degree, he judged that
the orifice, by which the contents of the tumor communicated with the
spinal canal, was but small. The prognosis, unfavorable from the nature
of the disease, was rendered still more so by the rapid increase of the
tumor, and by the highly attenuated state of the integuments which seemed
to be on the point of giving way. The parents of the child being anxious
to have something done for the cure of the disease, and being willing to
run the risk of an operation, Professor Gross decided to treat the case by
injections of iodine, according to the method proposed, and practiced
with such encouraging results by Professor Brainard, of Chicago.
On the nineteenth of October, a puncture was accordingly made in the
sound skin, about one inch and a quarter from the base of the tumor, by
means of a flat, curved needle, and the instrument having been carried sub-
cutaneously into the tumor, one drachm of its serous contents was allowed to
escape; a solution of one-eighth of a grain of iodine and one quarter of a
grain of iodide of potassium in one drachm of water was then injected
by means of a small syringe, provided with a delicate, curved nozzle. A
dressing of adhesive straps and collodion was applied over the puncture,
but being found insufficient to prevent leakage, the puncture was closed
by the twisted suture. The tumor was thoroughly painted over with
collodion. Three drops of tincture of opium were administered to the
patient, and directions were given to keep him lying on the face.
On the second day after the operation a small opening was discovered
at the top of the tumor, from which there was a slight drainage. The
child had had some convulsions during the previous night. To prevent
the escape of more fluid the tumor was covered with several successive
layers of cotton, painted over with collodion, until a sheath of about one-
eighth of an inch in thickness was formed.
On the morning of the next day the tumor appeared to be less tense.
The child had passed a comparatively comfortable night, and took the
breast well. A dose of castor-oil was ordered to keep its bowels soluble.
On the evening of the same day an escape of fluid from the tumor was
again noticed, and was checked by another application of cotton and col-
lodion. The dressing was, however, inadequate to control the discharge
permanently, and on the morning of the fourth day the tumor was found
leaking again; this time the fluid escaped from the point where the
twisted suture was applied. The child had suffered from convulsions
during the previous night; it had a haggard appearance, and seemed
much sunken. Cotton and collodion were applied as before.
On the fifth day after the operation, the tumor burst completely, all its
contents escaping suddenly through a large rent at its top. The child
died under severe convulsions.
The autopsy was made twenty-four hours after death. Only the tumor
was allowed to be examined. On laying the sac completely open, its
lining membrane was observed to be highly injected, and coated with a
thin layer of lymph, which in some places was softened and broken down.
The opening by which the cyst communicated with the spinal canal was
but of small size; it consisted of a cleft, about a quarter of an inch wide,
involving the arch of the fifth lumbar vertebra, the spinous process of
which was wanting. No nerve-twigs could be discovered passing out
from the spinal canal into the interior of the sac.
Case 2.—Charles Lehmann, three months of age, was brought to the
Clinic of the Jefferson Medical College, in October, 1859, on account of
a tumor over the lumbar region. The child’s health was excellent, and it
was perfectly well developed in every other respect. The tumor was
situated in the median line over the lower portion of the lumbar region,
projecting somewhat over the sacrum. It was globular, about as large as
a medium-sized billiard ball, translucent, fluctuating, and elastic. The
skin covering it was not discolored, but thin and shining, although by no
means as attenuated as in the case reported above. Upon pressure, the
size of the swelling was slightly diminished, but no fluctuation could be
perceived at the fontanels. The child moved its lower extremities con-
vulsively whenevever the tumor was touched with any degree of force.
At the time of birth the tumor was flabby, soft, and hardly half of its
present size. Its enlargement had taken place very gradually. The
child had the perfect use of its limbs, and all the functions of the body
were regularly performed. Considering the more advanced age of the
child, the smaller size and slower growth of the tumor, and the more
favorable condition of the integuments, this case seemed to be better
suited to the treatment by iodine than the preceding, and Professor
Gross, therefore, on the 19th of October, 1859, threw into the swell-
ing, the puncture having been made as in the other case, a drachm of
water, containing one-eighth of a grain of iodine and the fourth of a
grain of iodide of potassium. The puncture had to be closed by the
twisted suture, a very delicate cambric needle being used, and the whole
tumor was thoroughly coated with collodion. As in the other case, the
recumbent position on the abdomen was enjoined, and was carried out as
well as possible.
In the evening after the operation the child had slight convulsions,
which were relieved by three drops of laudanum. The operation was
repeated on the twenty-sixth of October, and again on the second of No-
vember, by which time considerable diminution in the volume of the tumor
had taken place. From the latter date to the twenty-third of December,
when the case terminated by death, the injections were thrown in every
fourth or fifth day, the quantity of iodine being increased to one-sixth of
a grain. Collodion was applied twice a day, and just before the patient
died the tumor had diminished two-thirds, leading Professor Gross to
suppose that the case might soon terminate in a cure. The child rarely
suffered from convulsions after the operations, and they always readily
yielded to a dose of castor-oil. Whenever there was pain, a few drops
of laudanum were administered, with a happy effect.
The child died in convulsions on the twenty-third of December, the
tumor having burst and discharged its contents. An autopsy was
made at the expiration of sixteen hours. The cavity of the sac was found
to be about one-third obliterated by coagulated lymph. The fissure
involved the posterior portion of the last lumbar vertebra and the first
piece of the sacrum, but the arachnoid on each side of the fissure was
covered with lymph, which caused the gap to be of smaller size than it
was originally. Had the patient lived a few weeks longer, a cure might
possibly have resulted.
Remarks.—On presenting these cases to his class, Professor Gross
made the following remarks : The two cases which at present engage our
attention exhibit a form of congenital malformation, known to patholo-
gists by the name of hydrorachitis, or spina bifida. It consists of a
fluid swelling, constituted by the membranes of the spinal cord, which are
distended and protrude backward through a congenital cleft of the ver-
tebral column.
This affection, which is closely analogous in its character to congenital
hydrocephalus, with which it is frequently associated, is attended with an
abnormal collection of fluid within the membranous sac of the spinal
column, from which it has received the name of hydrorachitis, or dropsy
of the spine. Whether this collection of fluid is owing to inflammation
of the membranes of the spinal cord, disturbances in the nutrition of the
part, or other causes operating during intra-uterine life, it is impossible
to say. The most common seat of the serous effusion is the sub arach-
noidean space, the fluid of which is thus augmented beyond its normal
quantity, and may amount from a few ounces to several pounds. In some
exceedingly rare cases the exudation has been found to occupy a space
between the dura mater and the bony walls of the vertebral column. The
fluid in hydrorachitis is generally of a thin, limpid character, slightly
saline in its taste, and almost uncoagulable. Sometimes, however, it is
glutinous, yellowish, sanguinolent, or contains flakes of lymph and par-
ticles of pus, appearances which are attributable to secondary processes of
an inflammatory character.
Although dropsy of the spine is accompanied, in the generality of cases,
by more or less extensive malformation of the vertebral column, it may
exist without any such malformation. In the latter case, the vertebral
column remaining in a normal condition, the disease may not give rise to
any outward deformity, or it may, as has been observed in some very rare
instances, cause a sac, formed by the membranes of the spinal marrow, to
protrude between two transverse processes from the canal, in the cervical
or lumbar region, and to effect a lodgment immediately underneath the
external integuments.
The malformation of the spine with which the affection is usually at-
tended, and from which it has received the name of spina bifida, consists in a
deficiency of the vertebral rings, owing to an arrest of ossification, and may
be compared to those defects which originate from a want of union of the
two halves of the foetus during utero-gestation, such, for instance, as
hare-lip and cleft palate. It may be arranged under the following heads :
1. Division of the entire vertebra, even of its body. 2. Partial or com-
plete absence of the lateral arches. 3. Perfect development of the lateral
arches with want of union at the median line. In the highest degree of
spina bifida the anomaly involves the whole length of the vertebral column,
and is then generally associated with extensive destruction of the spinal
marrow. In the more common cases, however, such as are likely to come
under the observation of the surgeon, the cleft in the spinal canal is but
partial, affecting usually the lumbar region, occasionally, however, the
dorsal, cervical, or even the sacral. The protrusion of the membranes of
the spinal marrow through this cleft generally takes place during foetal
life; in some instances it does not occur until some weeks or months
after birth. The tumor varies much in size, being sometimes so
small as to be hardly perceptible, at other times attaining the size of a
child’s head; a variation which seems to depend, in a great measure, upon
the smaller or greater extent of the cleft. Usually the swelling does
not exceed the size of an orange. In its form it is globular, ovoidal, or
pear-like, with a short and more or less constricted neck. The anatomical
composition of the walls of the tumor is variable; their outer layer is
generally constituted by skin, which is commonly very smooth, delicate,
and thin, sometimes, however, retaining its normal thickness, or becoming
red, rugose, and horny; their inner layers consist of the membranes of the
spinal cord. In other, more rare cases, the skin is entirely wanting, and
the walls are formed by the spinal membranes alone, which may be either
in their natural condition, or may be red, swollen, and thickened. The
contents of the tumor, consisting principally of the fluid described above,
communicate with the subarachnoidean fluid contained in the vertebral
canal. The swelling is usually formed of a single cyst, but there may be
several, as in the multilocular variety of ovarian dropsy. Virchow,
Glaser, and others describe, as congenital cystic hygroma of the sacral
region, a tumor which sometimes attains a very large size, but is still in
some cases curable by an operation; it is usually very complicated in its
structure, and is accompanied by a cleft or incomplete development of the
sacrum, but, in spite of it, does not generally communicate with the ver-
tebral canal.
In very slight cases of hydrorachitis, the spinal marrow and the nerves
originating from it are not all affected or changed. In many cases, how-
ever, the spinal marrow is flattened, and sometimes deviates from its ac-
customed route, being forced through the opening in the vertebrae, and
partially contained in the swelling. The nerves are always more or less
displaced; sometimes they are dragged out of the canal, and distributed
over the inner surface of the cyst in a plexiform manner. The textural
changes to which the spinal marrow is subject in hydrorachitis are
various; it is often very much softened, or converted into a thin, diffluent
mass; sometimes it has been found abnormally hard; sometimes it is not
as large as in the natural state. These pathological changes are in most
cases confined to those parts of the spinal cord which correspond with
the seat of the cleft in the vertebral column. In some cases of hydrora-
chitis the spinal marrow may suffer more or less from arrest of develop-
ment; instances are met with, though of rare occurrence, in which the
central canal existing in the spinal marrow during foetal life remains
open, and is distended by serous effusion. In the most serious cases the
spinal cord is in part, or in its entire length, wanting, either because it has
not been developed at all, or because it has been destroyed or absorbed by
the fluid collected in the subarachnoidean space; in the latter case, the
contents of the sac contain occasionally fragments of the spinal marrow
admixed with them.
Congenital hydrorachitis is, in the generality of cases, associated with
congenital hydrocephalus; the latter affection being usually proportionate
in degree to the former; in the highest forms of hydrorachitis, where
the spinal marrow is completely absent, the brain is also generally want-
ing. Other congenital defects, with which hydrorachitis may be compli-
cated, are hypospadias, extrophy of the bladder, imperforate anus, and
hare-lip. Club-foot and similar deformities of the lower extremities are
a very frequent complication. In some cases spina bifida is the only
lesion, and the rest of the body is in a perfectly normal condition.
It would be interesting to know whether the cleft of the vertebral
column is the primary, and the hydrorachitis the secondary affection, or
whether the reverse is the case. The fact that a moderate deficiency in
the vertebral rings, or a small cleft in the sacrum is sometimes found on
dissection in subjects without any signs of hydrorachitis having ever ex-
isted, that the protrusion of the spinal envelopes does occasionally not
take place until some time after birth, and that the tumor becomes larger
and larger the longer it continues, seem to speak in favor of the first
view; while the frequent coexistence of hydrocephalus with spina bifida,
and the circumstance that the one will subside greatly while the other in-
creases, and that plates of cartilage, constituting the laminae, are not rarely
found in the tissues covering the tumor, seem to indicate that the arrest
of ossification is owing to the distention of the sac of the spinal column
by the serous effusion. It is probable that in all cases, where the spinal
marrow is destroyed, or has suffered important changes in its structure
and course, a hydrorachitis was the primary affection; while in those
cases where there is a protrusion of the spinal membranes alone, and the
spinal marrow is in a normal condition, the lesion is secondary to the affec-
tion of the osseous parts.
The symptoms of hydrorachitis during life depend, principally, upon
the situation and size of the tumor, the condition of the spinal marrow,
and upon the complications which are present. The tumor, of which the
situation, size, form, and external appearance have already been de-
scribed, is either soft, flabby, and fluctuating, or it is full, hard, and
shining. If it is of large size, it is often transparent. At its base, the bony
margins of the cleft in the Vertebral column can sometimes be distinctly
felt. The swelling increases slightly during expiration, as well as crying
and defecation in the vertical position, and diminishes during inspiration
and rest; these symptoms, however, may be absent, if the communication
with the spinal canal is very small. When pressed upon, the tumor
gradually diminishes in volume, or completely recedes, but on removal of
the force, the fluid reaccumulates, and the part regains its previous bulk.
Pressure is painful when the external integuments or the inner wall of
the tumor is inflamed, or when the tumor contains spinal marrow and
nerves. In the latter case it may produce convulsions of the lower ex-
tremities. If the opening in the spinal canal is large, if hydrocephalus is
present, and the fluid contained in the sac communicates freely with the
cerebro-spinal fluid, pressure upon the tumor affects the fontanels, which
are rendered prominent, and may produce general convulsions, symptoms
of paralysis, and sopor. This effect of pressure will not, however, be
noticed, if the neck of the sac is very small. In all cases where the dis-
position of the spinal marrow and its connection with the tumor is of a
description that it may be easily irritated by pressure, by movements, and
other external influences, general convulsions are apt to occur, and there
may be contraction and rigidity of the lower extremities. When the
pathological changes from which the spinal marrow has suffered are very
considerable, or when there is great pressure upon it from fluid, the ex-
tremities may be paralyzed, cold, and emaciated, and there may be
palsy of the sphincter muscles of the bladder and rectum. In most
cases the general health of the children is seriously impaired; they be-
come feeble, pale, and emaciated, vomit frequently, digest badly, are liable
to suffer from inflammation of the urinary organs, and from ulceration of
the skin and excoriation, and are disturbed in their sleep by pain. Some
of the patients fall, a short time before death, into a state of stupor.
Sometimes, however, if the disease is developed but to a moderate degree,
it may remain without any influence upon the rest of the body.
Congenital hydrorachitis is nearly always fatal. In the highest degrees
of the disease, when the cleft implicates the whole length of the vertebral
column, and the spinal marrow and the brain are completely destroyed, or
when there is destruction of a considerable part of the spinal marrow, the
foetus loses its vitality at an earlier or later period of intra-uterine life, and
is prematurely expelled. If the child attains full development it may die
during labor in consequence of sudden rupture of the sac, or because the
large size of the tumor proves an impediment to the termination of labor.
If the children are born alive, they may die sooner or later in consequence
of the sudden escape of the contents of the tumor, the sac being opened
either by ulceration, accident, or design; death, under these circumstances,
is owing to convulsions from the pressure being taken off the brain in con-
sequence of the loss of the cerebro-spinal fluid. In other cases the fatal
termination is brought about by inflammation of the sac, which may extend
to the membranes of the spinal cord, and even of the brain, or by convul-
sions. When the case is associated with hydrocephalus, paralysis of the
inferior extremities, or involuntary discharge of the urine and feces, a
speedy death is likely to ensue. Few children, affected with hydrorachitis,
survive their birth longer than five or six months, while many perish in a
much shorter time. It is true, life has sometimes been sustained until the
age of puberty, and in one case, until the fifty-fifth year; but such instances
are altogether of an exceptional character.
In the treatment of hydrorachitis, the use of internal remedies is of no
avail, except to combat some of the intercurrent symptoms which may
arise. How little is gained by external surgical treatment, experience
has sufficiently shown. There is not the least prospect of success from
surgical interference, whenever the disease is complicated with a high de-
gree of hydrocephalus, or with other malformations of a serious character,
when there is more than one tumor present, when the swelling has a broad
base and is not covered by normal skin, when there are symptoms of the spi-
nal marrow being much affected, or having prolapsed together with nerves,
into the tumor. On the contrary, when the tumor has a narrow pedicle,
is small, painless, completely covered with sound skin, when it seems to
contain but little fluid, and is situated over the lower part of the ver-
tebral column, there is a reasonable hope that a cure may occasionally be
obtained.
Of the various modes of treatment devised for the cure of hydrorachitis,
that by compression is perhaps one of the safest and best. It may be ap-
plied by means of a cup-shaped truss, lined with an air-cushion, so as to
diffuse the pressure equally over the entire swelling; or the tumor may be
painted over repeatedly with collodion, and the action of the latter aided
by a common roller or adhesive straps. Several cases are reported in
which a cure was obtained by a combination of methodic pressure with
frequently repeated puncture; the latter is to be made subcutaneously
with a very fine trocar or bistoury, the opening being well closed imme-
diately afterwards, to prevent the introduction of air. Only a portion of
the fluid should, however, be drawn off at a time, as a sudden evacuation
of the tumor would be followed by the serious consequences already de-
scribed. Sir Astley Cooper, early in life, treated successfully a case of
cleft spine with compression alone, and, at a later period, succeeded in
curing two cases by combining this method with repeated punctures, as
proposed by Mr. Abernethy; in one of these cases the treatment was
carried on during eighteen months, the tumor having been punctured
thirty times before a cure was obtained.
The introduction of a seton, excision, or even simple incision of the
tumor are nearly always followed by speedy death, and should never be
attempted. The plan of tying the base of the tumor with a ligature,
suggested by Benjamin Bell, with a view of removing the swelling and
resisting the further propulsion of the spinal membranes, has been fol-
lowed, in those cases in which it has been tried, by so little success, that
there is no encouragement for its repetition. The patients died either
from convulsions immediately after the operation, or a short time after-
wards from an extension of the inflammation to the spinal cord and its
membranes. The application of the ligature can be proper only in those
very rare cases where the sac has a very narrow pedicle, and the aperture
of communication is exceedingly small, but even under these circumstances
the treatment by systematic compression, combined with repeated punc-
ture, would be preferable, as it is connected with less risk to the patient,
while offering at least equal chances of success. An instrument, resem-
bling somewhat an enterotome, has been invented, in order to compress the
base of the tumor, but has hardly any advantage over the ligature, upon
the principle of which it acts. Excision of the tumor after ligation of the
pedicle, and the application of the actual or potential cautery for the pur-
pose of exciting adhesion between the opposing surfaces of the sac, are pro-
cedures which are generally followed by a fatal result, and are by no
means to be recommended. The treatment of hydrorachitis by injection
of iodine, as practiced in the two cases reported above, has been applied
by different surgeons with various success. Professor Brainard of Chicago,
by whom this treatment was first instituted, gives (Chicago Medical
Journal, September, 1859, p. 517,) the result of ten cases treated in this
manner; they occurred partly in his own practice, partly in that of Dr.
Crawford, Chassaignac, Velpeau, and Nelaton. Of these ten cases, five,
it is alleged, were cured, three died, and in two the results remained un-
certain. The principal objection to this plan of treatment is, that it is
impossible to circumscribe the resulting inflammation sufficiently to pre-
vent it from spreading to the spinal marrow and its membranes.
In those cases of hydrorachitis, finally, in which surgical interference is
deemed improper, the tumor should be protected, by a proper contriv-
ance, from pressure, friction, and contact with the excretions; astringent
lotions, fomentations, and ointments may be tried, with a view of pro-
ducing thickening and shrinking of the walls of the sac, and tincture of
iodine may be applied in order to promote absorption of the contents of
the tumor.
III.—Foreign Body in the Right Bronchus; Tracheotomy; Removal
and Recovery. By John M. Adler, M.D., of Davenport, Iowa.
W. II., aged seven years, while playing with a piece of pipe-stem held
in the mouth, during an inhalation sucked it into the glottis. Severe
spasmodic cough followed, nearly suffocating him; and, while his mother
ran for medical aid, one of the neighbors, in making an effort to extricate
the foreign body with the fingers, forced it into the larynx, from which it
fell immediately into the trachea.
Various measures were at once resorted to with a view to its removal,
including the use of emetics, the introduction of the probang, inversion
of the body, and other means; and during a violent spasm of coughing, it
was supposed that the foreign body had been ejected from the air-passages
and swallowed. Continued dyspncea and suffocative cough, however,
subsequently gave unmistakable evidence that such was not the case.
On Saturday, the nineteenth of May, six days after the accident occurred,
I was called to see the patient, and found him half reclining on a couch,
breathing with great difficulty, pulse 140 to 160, countenance anxious,
skin hot and bathed in profuse perspiration. Careful auscultation at once
revealed the existence of the foreign body in the right bronchus, the
upper end projecting above the septum, and almost preventing the en-
trance of air into the left lung. There was nearly an entire absence
of respiratory sounds in the right lung, but occasionally a hoarse, hissing
noise was preceptible as the air was forced through the hole in the pipe-
stem or found its way past the obstruction. If inclined to the left side, the
dyspnoea was greatly increased and the passage of air into the lung of
that side almost entirely obstructed. When turned on the right side, the
symptoms were less urgent, the air passing into the left lung with greater
freedom. From the description of the piece of pipe-stem, given by the
parents, and the signs revealed by auscultation, I concluded that the posi-
tion of the foreign body was such as has been above stated. As repeated
attempts had been already made to remove it by inversion and succussion
of the body, it was at once decided that tracheotomy would be the only
chance for relief to the patient.
The consent of the parents being readily given, on the following morn-
ing, assisted by Drs. Fountain, Maxwell, Baker, and Tomson, the opera-
tion was performed. Chloroform was administered until perfect insensi-
bility was induced, and the head being thrown well back over the end of
the table, an incision, nearly two inches in length, was made from the cri-
coid cartilage toward the sternum. The muscles were carefully separated
along the raphe, and the isthmus of the thyroid gland, which was pro-
longed far down over the trachea, was divided to the extent of three or
four lines. The vessels were carefully held out of the way by blunt hooks,
and the trachea thoroughly bared to the extent of little over an inch.
There was no hemorrhage, except from the division of the thyroid body,
which was promptly checked by the application of Squibb’s solution of
persulphate of iron. The larynx being fixed by the forefinger of the
left hand, at the moment of expiration, with a narrow, sharp-pointed bis-
toury, four rings of the trachea were divided, the slit being fully an inch
in length. The breathing through the new opening soon became regular
and easy, and a slender pair of gullet forceps were carefully passed along
the trachea toward the right bronchus. The end of the forceps came im-
mediately in contact with the pipe-stem, grating against which, it slid
readily into the left bronchus. Several attempts were then made to get
hold of the pipe-stem, but failed, owing to the difficulty of expanding the
blades of the instrument sufficiently to grasp it, as well as from the stony
hardness and the roundness of the foreign substance. A partial hold
upon it was several times effected, but the blades of the forceps as repeat-
edly slipped from it. Various other instruments were then used, but
without success. The trachea was quite tolerant of their introduction,
though during the whole time of their remaining in it continual cough-
ing interfered much with the precise manipulation required to grasp and
remove the foreign body.
All our efforts at extraction having failed, it was determined to leave
the patient awhile, and to trust to the efforts of nature in loosening the
foreign body by suppuration, and its being thus spontaneously expelled
or rendered more easy of removal. He was therefore left until the next
morning, with proper directions to the parents as to treatment.
In the morning his condition was much more alarming; pulse 150 to
160; mucous rales throughout the left lung; the right engorged, dull on
percussion, and the entrance of the air into it apparently quite stopped;
had passed a very restless night, and was much prostrated.
Assisted by Dr. Maxwell, efforts were again made to get hold of the
foreign substance, which was easily accomplished by means of a wire
hook, but we failed to remove it. The case appeared almost hopeless,
but as it was evident that unless the obstruction was relieved, the patient
would certainly die, it was determined to make another and final attempt
in the evening.
About 6 p.m., assisted by Dr. Tomson, the patient was placed in the
proper position, and similar attempts at extraction again made, which
finally resulted in success. The instrument used was simply a loop of
iron wire. The method of employing it will be readily understood by refer-
ence to the familiar process of removing, with a loop of twine or wire, a
cork from an empty bottle. The loop was passed along and beyond the
pipe-stem and then withdrawn, bringing the pipe-stem caught in the loop,
which passed around the end, the sides of the loop at the same time
steadying the foreign substance in its passage through the trachea. The
pipe-stem measurejl one inch in length, and nearly five-sixteenths of an
inch in diameter.
Immediate relief of the dyspnoea resulted; the slit in the trachea was
closed with adhesive strips, and directions given to remove them in case
it should become much obstructed during the night by the profuse mucous
secretion from the lungs. Some light broth and a full opiate were admin-
istered. On the following day the wound was closed with four hare-lip
sutures.
Perfect quiet, with strict antiphlogistic treatment, was followed for sev-
eral days. Antimonials, with the tincture of veratrum viride, and the
application of a large blister on the right side, soon successfully controlled
the resulting inflammation of the lungs, and the patient made a speedy
recovery.
In fourteen days from the time of the operation the wound was per-
fectly healed, with scarcely a perceptible cicatrix, and the little fellow was
running about with his playmates as well as ever.
				

